# DeepLBCEPred: A Bi-LSTM and multi-scale CNN-based deep learning method for predicting linear B-cell epitopes

**DOI:** 10.3389/fmicb.2023.1117027

**Published:** 2023-02-22

**Authors:** Yue Qi, Peijie Zheng, Guohua Huang

**Affiliations:** School of Information Engineering, Shaoyang University, Shaoyang, Hunan, China

**Keywords:** epitope, B-cell, CNN, LSTM, protein sequence

## Abstract

The epitope is the site where antigens and antibodies interact and is vital to understanding the immune system. Experimental identification of linear B-cell epitopes (BCEs) is expensive, is labor-consuming, and has a low throughput. Although a few computational methods have been proposed to address this challenge, there is still a long way to go for practical applications. We proposed a deep learning method called DeepLBCEPred for predicting linear BCEs, which consists of bi-directional long short-term memory (Bi-LSTM), feed-forward attention, and multi-scale convolutional neural networks (CNNs). We extensively tested the performance of DeepLBCEPred through cross-validation and independent tests on training and two testing datasets. The empirical results showed that the DeepLBCEPred obtained state-of-the-art performance. We also investigated the contribution of different deep learning elements to recognize linear BCEs. In addition, we have developed a user-friendly web application for linear BCEs prediction, which is freely available for all scientific researchers at: http://www.biolscience.cn/DeepLBCEPred/.

## 1. Introduction

B cells are a class of leukocytes that are subtypes of lymphocytes in the immune system (Murphy and Weaver, 2012). B cells respond to foreign antigens by producing B-cell receptors that bind to the antigen (Murphy and Weaver, 2012). The sites where an antigen binds to an antibody are called epitopes (also known as antigenic determinants), which are specific pieces of the antigen. According to the structure and interaction with antibodies, epitopes can be grouped into conformational and linear epitopes (Huang and Honda, 2006). Conformational epitopes consist of discontinuous amino acid residues, and linear epitopes comprise contiguous amino acid residues. Identification of B-cell epitopes (BCEs) is not only essential for understanding the mechanisms of antigen–antibody interactions but also for vaccine design and therapeutic antibody development (Sharon et al., 2014; Shirai et al., 2014).

In contrast to labor-intensive and costly experimental methods, computational identification is cheap and high-throughput (Peng et al., 2022; Shen et al., 2022; Tian et al., 2022). Over the past decades, no less than 10 computational methods for predicting BCEs have been created (El-Manzalawy et al., 2008a, 2017; Ansari and Raghava, 2010; El-Manzalawy and Honavar, 2010; Jespersen et al., 2017; Ras-Carmona et al., 2021; Sharma et al., 2021; Alghamdi et al., 2022). The sequence is the simplest manifestation of protein but is pivotal for structure and function formation, and thus, the sequence compositions were frequently employed as a factor to identify BCEs (Chen et al., 2007; Singh et al., 2013). The sequence composition included but was not limited to the physico-chemical profile (Ansari and Raghava, 2010), amino acid pair propensities (Chen et al., 2007; Singh et al., 2013), the composition–transition–distribution (CTD) profile (El-Manzalawy et al., 2008b), the tri-peptide similarity and propensity score (Yao et al., 2012), and subsequence kernel (El-Manzalawy et al., 2008a). The sequence composition might not represent all characteristics of the BCEs because it lacks position-related or order-related information. Other representations such as evolutionary features (Hasan et al., 2020) and structural features (Zhang et al., 2011) were explored as a determinant for identifying BCEs. There are three key factors responsible for the accuracy of identifying BCEs: the number and quality of BCEs served as training samples, representations, and learning algorithms. Jespersen et al. (2017) used the BCEs derived from crystal structures as the training set to improve prediction accuracy. Informative representations for BCEs are highly desirable but are too difficult to achieve in practice. Exploring new representations or combining various existing representations are two inevitable selections. Hasan et al. (2020) employed a non-parametric Wilcoxon rank-sum test to explore informative representations, while Chen et al. (2007) proposed a new amino acid pair antigenicity scale to represent BCEs. New representations are not always more informative than existing representations, and searching for an optimal combination of representations is both time-consuming and not always efficient. The learning algorithm is another factor to consider when developing methods for BCEs recognition, which plays equivalent roles with representations. The effectiveness of the learning algorithm might be associated with representations, that is, algorithms are representation-specific. It is ideal to search for an optimal scheme between algorithms and representations to enhance predictive performance. For example, Manavalan et al. (2018) explored six machine learning algorithms as well as appropriate representations and proposed an ensemble learning algorithm for linear BCEs recognition. Recently, deep learning is emerging as the next-generation artificial intelligence, exhibiting powerful learning ability. Deep learning has made a great breakthrough in areas such as image recognition (Krizhevsky et al., 2017) and mastering Go game as well as protein structure prediction (Silver et al., 2017; Cramer, 2021; Du et al., 2021; Jumper et al., 2021). To the best of our knowledge, there are more than three deep learning-based methods for predicting BCEs (Liu et al., 2020; Collatz et al., 2021; Xu and Zhao, 2022). Liu et al. demonstrated remarkable superiority of deep learning over traditional machine learning methods by cross-validation. Collatz et al. (2021) proposed a bi-directional long short-term memory (Bi-LSTM)-based deep learning method (called EpiDope) to identify linear BCEs. The EpiDope showed better performance in empirical experiments. Inspired by this, we improved EpiDope by adding a multi-scale convolutional neural networks (CNNs) to promote representation.

## 2. Dataset

We utilized the same benchmark datasets as BCEPS (Ras-Carmona et al., 2021) to evaluate and compare our proposed method with state-of-the-art methods. These datasets were initially extracted from the Immune Epitope Database (IEDB) (Vita et al., 2015, 2019), a repository of experimentally validated B- and T-cell epitopes (Vita et al., 2010). Ras-Carmona et al. (2021) constructed a nonredundant dataset BCETD_555_ as the training set, which includes 555 sequences of BCEs and 555 sequences without BCEs. The BCEs in BCETD_555_ consisted of linearized conformational B-cell epitopes (Ras-Carmona et al., 2021), obtained from the tertiary structure of the antigen–antibody complexes (Ras-Carmona et al., 2021). Ras-Carmona et al. (2021) used CD-HIT (Li and Godzik, 2006) to reduce sequence redundancy by deleting epitope sequences with more than 80% homology. Two independent testing sets were downloaded directly from https://www.mdpi.com/article/10.3390/cells10102744/s1 (Ras-Carmona et al., 2021): one set is the ILED_2195_ dataset containing 2,195 sequences of linear BCEs and 2,195 sequences of non-BCEs and another set is the IDED_1246_ dataset containing 1,246 sequences of BCEs and 1,246 sequences of non-BCEs. The ILED_2195_ dataset and the IDED_1246_ dataset were retrieved from the experimental B-cell epitope sequences retrieved from the IEDB database (Vita et al., 2015, 2019). All non-BCE sequences were extracted randomly from the same antigens as the BCEs.

## 3. Method

**Figure 1:**
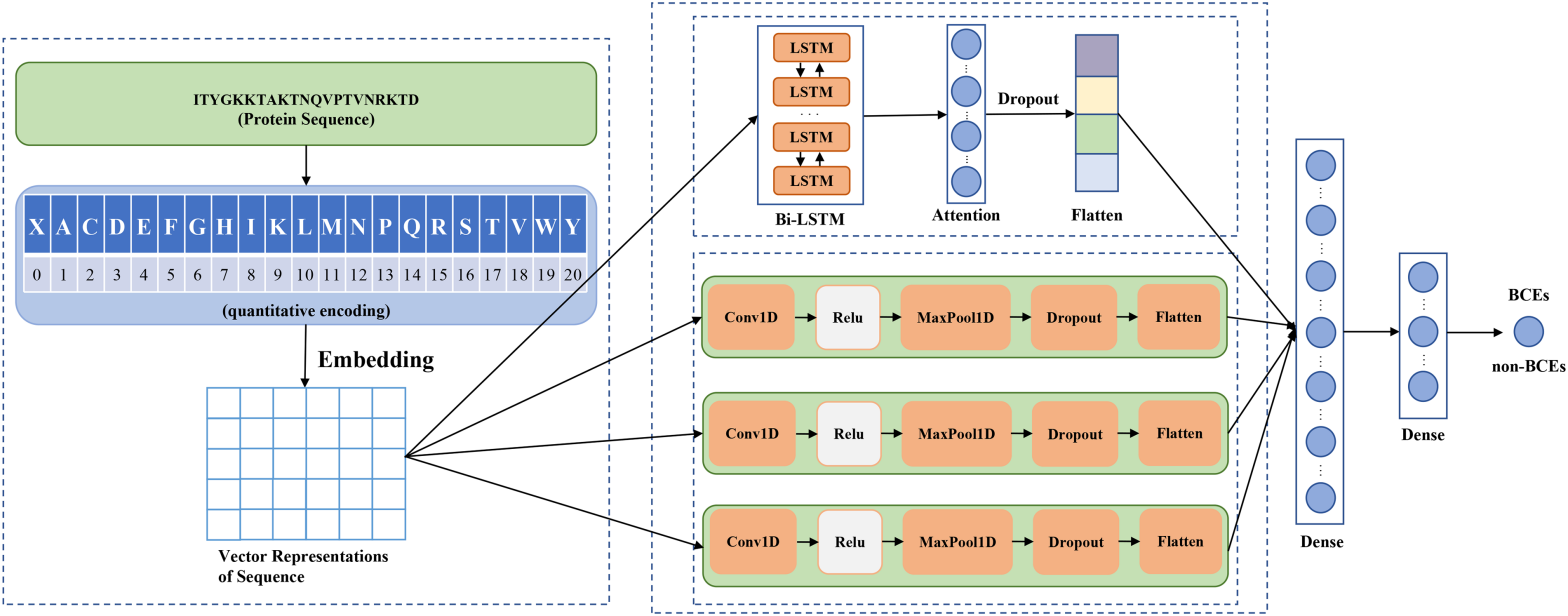
Schematic diagram of DeepLBCEPred.

Figure 1 showed the schematic diagram of the proposed method DeepLBCEPred, which mainly consists of input, quantitative coding, embedding, feature extraction, and classification. Inputs are protein primary sequences that comprise 20 amino acid characters. For any sequences of less than a given length, we added the corresponding number of special characters ‘X’ at the end of it. Inputs were 21-character text sequences. The character sequence must be converted into an integer sequence by quantization coding using a conversion table (Table 1) so that the integer sequence can be embedded in a continuous vector using an embedding layer. Feature extraction includes two paralleling parts, one consisting mainly of the Bi-LSTM (Schuster and Paliwal, 1997) layer followed by a feed-forward attention layer (Raffel and Ellis, 2015) and another comprising multi-scale CNNs. Bi-LSTM (Schuster and Paliwal, 1997) was intended to extract the contextual semantics of the sequences, while the feed-forward attention (Raffel and Ellis, 2015) was intended to promote the semantic representation of protein sequences. CNNs at different scales reflect the representation of protein sequences at different scales. We used three different scale CNNs for extracting multi-scale features of sequences. The classification includes three fully connected layers, where the first has 64 neurons, the second has nine neurons, and the third has one neuron, which represents the probabilities of predicting inputs as BCEs.

**Table 1:** Conversion between amino acid and integer.

X	A	C	D	E	F	G	H	I	K	L	M	N	P	Q	R	S	T	V	W	Y
0	1	2	3	4	5	6	7	8	9	10	11	12	13	14	15	16	17	18	19	20

### 3.1. Bi-LSTM

Long short-term memory (LSTM) (Hochreiter and Schmidhuber, 1997) is a specific type of recurrent neural network (RNN). Long short-term memory is capable of learning semantic relationships between long-distance words (Hochreiter and Schmidhuber, 1997). LSTM acts as a conveyor belt since it runs directly along the entire chain with only a few linear interactions (Hochreiter and Schmidhuber, 1997). At the heart of the LSTM is the cell state, which allows information to flow selectively by gate mechanisms (Hochreiter and Schmidhuber, 1997). There are three common gates: forget gate, input gate, and output gate. The forget gate is to determine how much information flows into the next cell state. The forget gate uses a sigmoid function to map the hidden state and input variables into a number between 0 and 1. While 1 represents all information to pass completely, 0 indicates that no information is passing through. The question of how much information is added to the state cell is determined jointly by the input gate and the candidate cell state. The hidden state is updated jointly by the cell state and the output gate. To capture bidirectional dependency between words, we used Bi-LSTM (Schuster and Paliwal, 1997) to refine the semantics.

### 3.2. Feed-forward attention

Attention mechanisms have received increasing attention from the deep learning community due to better interpretability. Over the past 5 years, many attention mechanisms have been proposed to facilitate the interpretation of representations, such as well-known self-attention (Vaswani et al., 2017), feed-forward attention (Raffel and Ellis, 2015), external attention (Guo et al., 2022), and double attention (Chen et al., 2018). The attention mechanism is a scheme for assigning weights to different parts. Here, we employed feed-forward attention (Raffel and Ellis, 2015) for improving semantic representation. The attention weight was computed by


(1) $$\;\alpha_{t}=\frac{\exp\left(e_{t}\right)}{\sum_{k=1}^{T}\exp\left(e_{k}\right)}\;$$

where$\;e_{t}=a\left(h_{t}\right)$.$\;h_{t}$ denoted the hidden state at the time step *t* in the Bi-LSTM and a was the learnable parameter. The output was computed by


(2) $$\;c=\overset{T}{\underset{t=1}{\sum }}\alpha_{t}h_{t}\;$$

### 3.3. Multi-scale CNNs

CNNs are one of the most popular machine learning algorithms and thus have extensively been applied for image recognition. CNNs are mainly comprised of two elements: a convolutional layer and a pooling layer. At the heart of the CNNs is convolutional operation, which is to multiply the convolutional kernel by the receptive field in an element-wise manner and then sum them up. The convolution operation is accompanied by the activation function that produces a non-linear transformation. The activation function is associated with the efficiency and effectiveness of CNNs to a certain extent, and thus, selecting the appropriate activation function is critical to promote the performance of CNN. The commonly used activation function includes sigmoid, tanh, and rectified linear unit (ReLu). The convolutional kernel slides along the input to convolve with the receptive field to generate different feature maps. The convolutional kernel is shared by all the receptive fields in the same input and is the learnable parameter. The size of the convolutional kernel determines the different-scale characterization of the input. The larger size convolutional kernel reflects the global information, and the smaller size convolutional kernel discovers the local structure. To capture multi-scale characterization, we used multi-scale CNNs. The pooling layer is a sub-sampling operation, which reduces the dimensionality of the representation and thus speeds up the calculation. The pooling includes max, average, overlapping, and spatial pyramid pooling (Wang et al., 2012; He et al., 2015; Khan et al., 2020). The dropout layer is used to randomly drop out some connections with a given probability to reduce computation and avoid overfitting (Hinton et al., 2012).

### 3.4. Fully connected layer

The fully connected layer is similar to the hidden layer in the multilayer perceptron where each neuron is linked to all the neurons in the previous layer. The outputs of the attention layer and the CNNs are of more than one dimension and, therefore, must be converted into one dimension to link to the fully connected layer. We used the flattened layer to bridge the fully connected layers and the non-fully connected layers. The flattened layers do not have any learnable parameters, and its actual task is to transform the shape of the data. We used three fully-connected layers. The first fully connected layer contains 64 neurons, the second contains 9 neurons, and the third contains only 1 neuron, which represents the probabilities of identifying inputs as BCEs.

## 4. Metrics

This is a binary classification question. The commonly used evaluation indices, namely, sensitivity (Sn), specificity (Sp), accuracy (ACC), and Matthews correlation coefficient (MCC), were employed to assess performance. Sn, Sp, ACC, and MCC were defined as follows:


(3) $$\;\mathrm{Sn}=\frac{\mathrm{TP}}{\mathrm{TP}+\mathrm{FN}}\;$$

(4) $$\;\mathrm{Sp}=\frac{\mathrm{TN}}{\mathrm{TN}+\mathrm{FP}}\;$$

(5) $$\;\mathrm{ACC}=\frac{\mathrm{TP}+\mathrm{TN}}{\mathrm{TP}+\mathrm{FP}+\mathrm{TN}+\mathrm{FN}}\;$$

(6) $$\;\mathrm{MCC}=\frac{\mathrm{TP}\times \mathrm{TN}-\mathrm{FP}\times \mathrm{FN}}{\sqrt{\left(\mathrm{TP}+\mathrm{FN}\right)\times \left(\mathrm{TN}+\mathrm{FN}\right)\times \left(\mathrm{TP}+\mathrm{FP}\right)\times \left(\mathrm{TN}+\mathrm{FP}\right)}}\;$$

where TP stands for the number of correctly predicted BCEs, TN stands for the number of correctly predicted non-BCEs, FP stands for the number of the non-BCEs, which were in reality non-BCEs but were erroneously predicted as BCEs, and FN stands for the number of the BCEs, which were in reality BCEs but were erroneously predicted as non-BCEs. Sn, Sp, and ACC lie between 0 and 1. The more the value is, the better performance there is. MCC considers not only TP and TN but also FP and FN and thus is generally viewed as a better measure for imbalanced datasets. MCC ranges from −1 to 1. An MCC of 1 implies perfect prediction, 0 implies random prediction, and − 1 implies inverse prediction.

## 5. Results

Protein sequences of BCEs are of variable length, which is not favorable for subsequent sequence embedding. Therefore, we had to standardize the length of all BCEs sequences. The maximum length of BCEs sequences is 25, the average length is 16, and the minimum length is 11. We used 20% of the training BCEs in the training set to validate the effect of sequence length on the predictive performance. As listed in Table 2, the maximum length reached the best performance, followed by the average length and then the minimum length. Therefore, we uniformed all the sequences into a fixed length of 25.

**Table 2:** Performance over the various sequence length.

Sequence length	Sn	Sp	ACC	MCC
11(minimum)	0.64	0.78	0.70	0.42
16(average)	0.74	0.73	0.73	0.47
25(Maximum)	0.80	0.74	0.77	0.54

Different scales reflect different scale characterization of the sequences. In this study, we used multi-scale CNNs. The combination of multi-scale CNNs is an optimal issue. To date, there is no scientific theory on how to effectively combine CNNs of different scales. In most cases, it relies on experience, especially experimental performances, to make choice. We investigated the effects of different scale combinations on the proposed method. The size of each scale ranged from 7 to 15 with a step size of 2. We used holdout to examine the performance. In the holdout, 80% was used to train the DeepLBCEPred and the remaining 20% was used to test the trained DeepLBCEPred, and the performance is presented in Table 3. When three scales of CNNs were set to 11, 13, and 15, respectively, the DeepLBCEPred reached the best ACC and the best MCC. Therefore, we set three scales to 11, 13, and 15, respectively.

**Table 3:** Performance of different scale combinations.

Scale 1	Scale 2	Scale 3	Sn	Sp	ACC	MCC
7	9	11	0.79	0.58	0.69	0.38
7	9	13	0.61	0.84	0.72	0.46
7	9	15	0.86	0.55	0.72	0.43
7	11	13	0.70	0.81	0.75	0.50
7	11	15	0.75	0.68	0.72	0.43
7	13	15	0.63	0.80	0.71	0.43
9	11	13	0.72	0.81	0.76	0.53
9	11	15	0.71	0.70	0.70	0.40
9	13	15	0.78	0.73	0.76	0.51
11	13	15	0.80	0.74	0.77	0.54

## 6. Discussion

### 6.1. Comparison with existing models

As mentioned previously, many computational methods, including BepiPred (Larsen et al., 2006; Jespersen et al., 2017), LBtope (Singh et al., 2013), IBCE-EL (Manavalan et al., 2018), LBCEPred (Alghamdi et al., 2022), and BCEPS (Ras-Carmona et al., 2021), have been developed for BCEs prediction over the recent decades. We extensively compared the DeepLBCEPred with those methods by conducting 10-fold cross-validation on the BCETD_555_ and independent tests on both ILED_2195_ and IDED_1246_. The 10-fold cross-validation divides BCETD_555_ into 10 parts in equivalent or approximately equivalent size, with one part used to test the trained DeepLBCEPred by the other nine parts. The process is repeated 10 times. When this process is over, each sample is used only one time for testing the model and nine times for training the model. The independent test is to use ILED_2195_ or IDED_1246_ to test the DeepLBCEPred trained by BCETD_555_. Table 4 lists their performance comparisons in 10-fold cross-validation. Compared to BCEPS, DeepLBCEPred increased ACC by 0.02, Sn by 0.05, and MCC by 0.03.

**Table 4:** Ten-fold cross-validation results of DeepLBCEPred.

Ten-fold cross-validation	Sn	Sp	ACC	MCC
1	0.82	0.71	0.77	0.54
2	0.75	0.73	0.74	0.48
3	0.73	0.79	0.76	0.51
4	0.85	0.70	0.77	0.56
5	0.69	0.82	0.76	0.52
6	0.88	0.62	0.75	0.51
7	0.77	0.82	0.79	0.59
8	0.75	0.80	0.77	0.55
9	0.70	0.82	0.76	0.52
10	0.86	0.73	0.79	0.59
Ten-fold cross-validation (Mean)	0.78	0.75	0.77	0.54
BCEPS (Ras-Carmona et al., 2021)	0.73	0.78	0.75	0.51

We compared DeepLBCEPred with five state-of-the-art algorithms by independent tests: BepiPred (Larsen et al., 2006; Jespersen et al., 2017), LBtope (Singh et al., 2013), LBCEPred (Alghamdi et al., 2022), IBCE-EL (Manavalan et al., 2018), and BCEPS (Ras-Carmona et al., 2021). The LBCEPred is a newly developed method for predicting linear BCEs (Alghamdi et al., 2022). We uploaded two independent datasets to the LBCEPred webserver which are available at http://lbcepred.pythonanywhere.com/pred for prediction. All the predictive performances are listed in Tables 5 and 6. The DeepLBCEPred obtained a distinct superiority in ACC as well as MCC over BepiPred (Larsen et al., 2006; Jespersen et al., 2017), LBtope (Singh et al., 2013), LBCEPred (Alghamdi et al., 2022), and IBCE-EL (Manavalan et al., 2018). On the ILED_2195_ independent dataset, the DeepLBCEPred exceeded the IBCE-EL by 0.16 of ACC as well as 0.33 of MCC, the LBtope by 0.17 of ACC as well as 0.35 of MCC, the BepiPred by 0.31 of ACC as well as 0.63 of MCC, and the LBCEPred by 0.15 of ACC as well as 0.31 of MCC. On the IDED_1246_ independent dataset, the DeepLBCEPred exceeded the IBCE-EL by 0.14 of ACC as well as 0.26 of MCC, the LBtope by 0.10 of ACC as well as 0.21 of MCC, the BepiPred by 0.19 of ACC as well as 0.39 of MCC, and the LBCEPred by 0.15 of ACC as well as 0.29 of MCC. Compared with the BCEPS (Ras-Carmona et al., 2021), the DeepLBCEPred still has a slight advantage in ACC as well as MCC. The DeepLBCEPred increased ACC by 0.04 and MCC by 0.08 over the ILED_2195_, and MCC by 0.01 over the IDED_1246_.

**Table 5:** Comparison with existing models on the ILED_2195_ independent dataset.

Model	Sn	Sp	ACC	MCC
IBCE-EL (Manavalan et al., 2018)	0.64	0.33	0.48	−0.04
LBtope (Singh et al., 2013)	0.36	0.58	0.47	−0.06
BepiPred (Jespersen et al., 2017)	0.24	0.43	0.33	−0.34
LBCEPred (Alghamdi et al., 2022)	0.74	0.24	0.49	−0.02
BCEPS (Ras-Carmona et al., 2021)	0.50	0.71	0.60	0.21
DeepLBCEPred	0.56	0.73	0.64	0.29

**Table 6:** Comparison with existing models on the IDED_1246_ independent dataset.

Model	Sn	Sp	ACC	MCC
IBCE-EL (Manavalan et al., 2018)	0.86	0.20	0.53	0.09
LBtope (Singh et al., 2013)	0.40	0.74	0.57	0.14
BepiPred (Jespersen et al., 2017)	0.42	0.52	0.48	−0.04
LBCEPred (Alghamdi et al., 2022)	0.79	0.26	0.52	0.06
BCEPS (Ras-Carmona et al., 2021)	0.63	0.71	0.67	0.34
DeepLBCEPred	0.60	0.75	0.67	0.35

### 6.2. Ablation experiments

Over the past decades, many basic structural units such as CNN, LSTM (Hochreiter and Schmidhuber, 1997), and self-attention (Vaswani et al., 2017) have been developed for deeper neural networks. Different units play different roles in characterizing studied objects. For instance, the CNN does well in refining local structure and Bi-LSTM (Schuster and Paliwal, 1997) in capturing long-distance dependency between words, while the self-attention emphasizes the key relationship of words. We investigated the contribution of a single individual to predicting BCEs by removing the corresponding part from the DeepLBCEPred. For the investigation, we performed independent tests after, respectively, removing (a) Bi-LSTM; (b) scale 1 in multi-scale CNNs; (c) scale 1 and scale 2 in multi-scale CNNs; (d) multi-scale CNNs; and (e) attention mechanism. As shown in Tables 7 and 8, the removal of these parts leads the performance to decrease. Deleting Bi-LSTM causes Sp to significantly reduce.

**Table 7:** Comparison of five ablation experiments on the ILED_2195_ independent dataset.

Ablation experiments	Sn	Sp	ACC	MCC
delete Bi-LSTM	0.69	0.53	0.61	0.22
delete scale 1	0.56	0.70	0.63	0.26
delete scale 1_2	0.53	0.68	0.60	0.21
delete Multi-scale CNN	0.45	0.71	0.58	0.17
delete Attention mechanism	0.55	0.66	0.60	0.21
DeepLBCEPred	0.56	0.73	0.64	0.29

**Table 8:** Comparison of five ablation experiments on the IDED_1246_ independent dataset.

Ablation experiments	Sn	Sp	ACC	MCC
delete Bi-LSTM	0.79	0.55	0.67	0.35
delete scale 1	0.62	0.70	0.66	0.31
delete scale 1_2	0.66	0.70	0.68	0.36
delete Multi-scale CNN	0.61	0.73	0.67	0.35
delete Attention mechanism	0.68	0.66	0.67	0.35
DeepLBCEPred	0.60	0.75	0.67	0.35

### 6.3. t-distributed stochastic neighbor embedding (t-SNE) visualization

**Figure 2:**
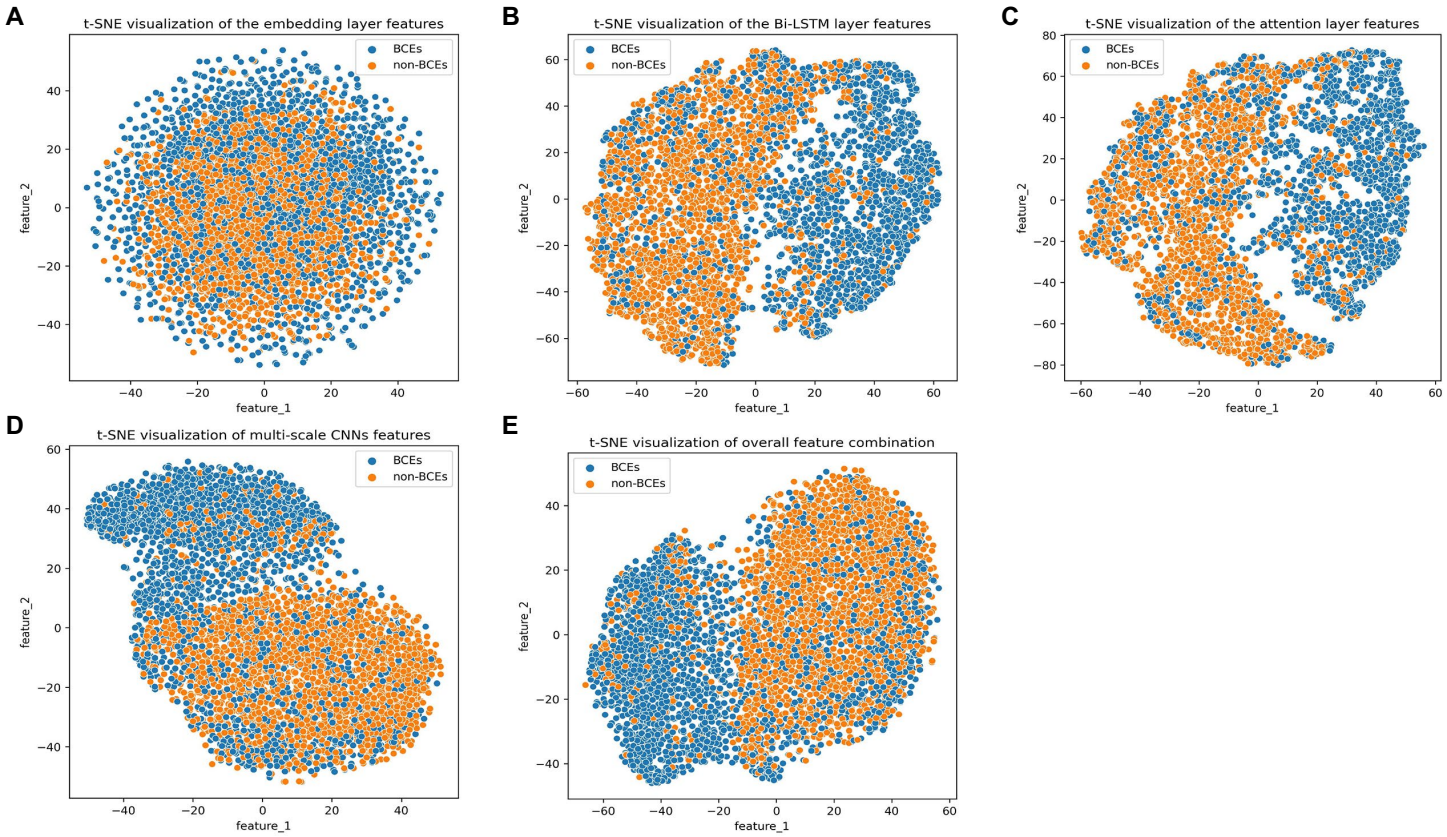
t-SNE visualization of outputs of **(A)** the embedding layer, **(B)** the Bi-LSTM layer, **(C)** the attention layer, **(D)** the multi-scale CNNs, and **(E)** overall combination.

We investigated the discriminative power of the representation captured by different layers in the DeepLBCEPred. We used the t-SNE (Van der Maaten and Hinton, 2008) to plot a scattering diagram of the first two components in the ILED_2195_ dataset. The initial embedding was highly indistinguishable. The representations output by multi-scale CNNs and Bi-LSTM were significantly distinguishable. The feed-forward attention improved representations to a tiny extent. The overall combined representations promoted discriminative ability, demonstrating the ability to distinguish between BCEs and non-BCEs from a representational perspective (Figure 2).

### 6.4. Deep learning community due to better interpretability web server

**Figure 3:**
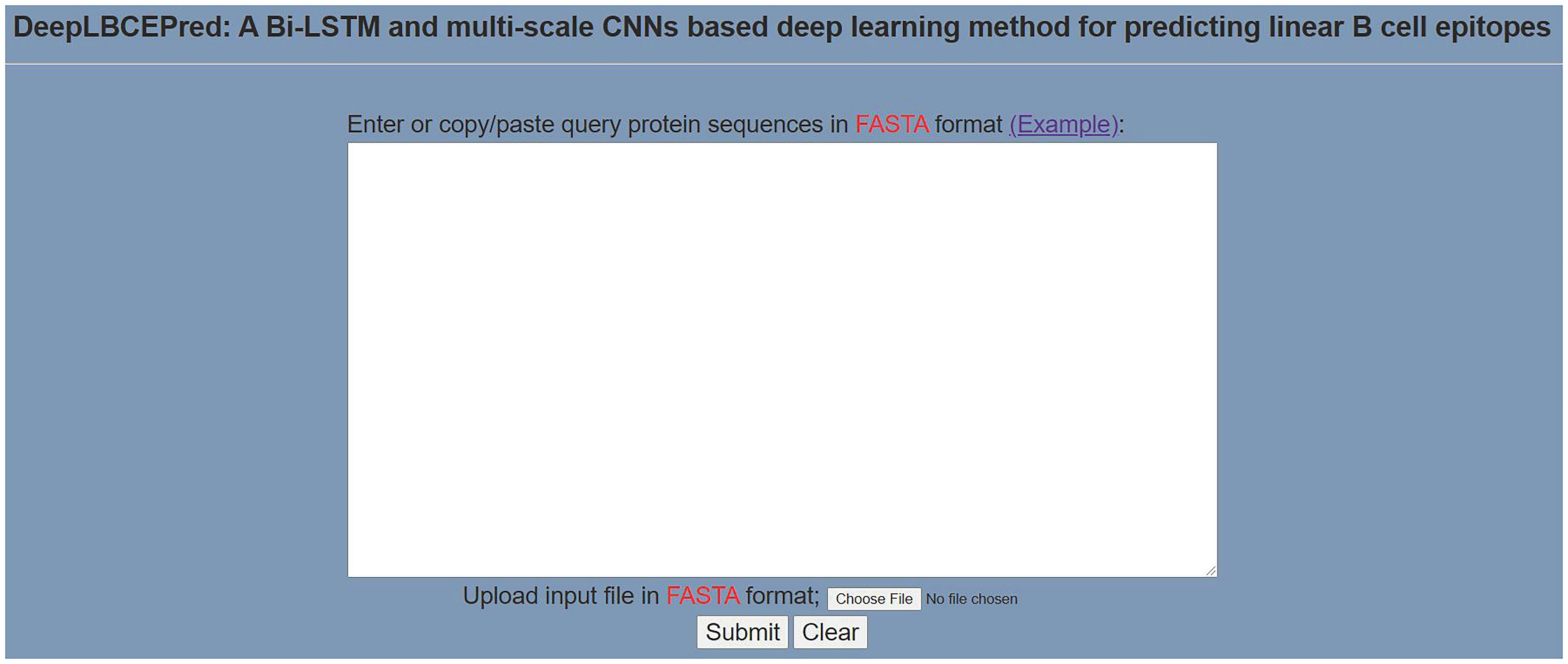
Prediction page of the web server.

To help researchers use DeepLBCEPred more easily, we have exploited a user-friendly web server, which is available at: http://www.biolscience.cn/DeepLBCEPred/. As shown in Figure 3, after the user writes a sequence in the text box or uploads a sequence file and clicks “Submit,” the page will display the final prediction result. It is worth noting that only the sequence in FASTA format is allowed, and the input sequence must consist of the characters in “ACDEFGHIKLMNPQRSTVWY.” Otherwise, it will prompt Format Error. To clear the contents of the text box, click “Clear.” Click “Example” to see a sample. The dataset used in this study can be downloaded from the bottom left corner of the page.

## 7. Conclusion

B-cell epitopes play critical roles in antigen–antibody interactions and vaccine design. Identification of BCEs is a key foundation for understanding BCEs functions. In the article, we developed a deep learning-based method DeepLBCEPred to predict linear BCEs. The DeepLBCEPred is an end-to-end method that takes protein sequence as input and directly outputs decisions about BCEs. On the benchmark datasets, DeepLBCEPred reached state-of-the-art performance and was implemented as a user-friendly web server for ease of use.

## Data availability statement

The original contributions presented in the study are included in the article/Supplementary material, further inquiries can be directed to the corresponding author.

## Author contributions

YQ conducted experiments, analysis, and wrote the original manuscript. PZ conducted experiments and developed the software. GH conceived the methodology, supervised the project and revised the manuscript. All authors contributed to the article and approved the submitted version.

## Funding

This work is supported by Hunan Province Natural Science Foundation of China (2022JJ50177), by Scientific Research Fund of Hunan Provincial Education Department (21A0466), and the Shaoyang University Innovation Foundation for Postgraduate (CX2021SY037).

## Conflict of interest

The authors declare that the research was conducted in the absence of any commercial or financial relationships that could be construed as a potential conflict of interest.

## Publisher’s note

All claims expressed in this article are solely those of the authors and do not necessarily represent those of their affiliated organizations, or those of the publisher, the editors and the reviewers. Any product that may be evaluated in this article, or claim that may be made by its manufacturer, is not guaranteed or endorsed by the publisher.

## References

[ref1] Alghamdi W. Attique M. Alzahrani E. Ullah M. Z. Khan Y. D. (2022). LBCEPred: a machine learning model to predict linear B-cell epitopes. Brief. Bioinform. 23:bbac035. doi: 10.1093/bib/bbac035, PMID: 35262658

[ref2] Ansari H. R. Raghava G. P. S. (2010). Identification of conformational B-cell epitopes in an antigen from its primary sequence. Immunome Res. 6, 6–9. doi: 10.1186/1745-7580-6-6, PMID: 20961417 PMC2974664

[ref3] Chen Y. Kalantidis Y. Li J. Yan S. Feng J. (2018). “A^2-nets: double attention networks” in Advances in Neural Information Processing Systems. eds. Bengio S. Wallach H. Larochelle H. Grauman K. Cesa-Bianchi N. Garnett R. Neural Information Processing Systems Foundation, Inc. (NeurIPS).

[ref4] Chen J. Liu H. Yang J. Chou K.-C. (2007). Prediction of linear B-cell epitopes using amino acid pair antigenicity scale. Amino Acids 33, 423–428. doi: 10.1007/s00726-006-0485-9, PMID: 17252308

[ref5] Collatz M. Mock F. Barth E. Hölzer M. Sachse K. Marz M. (2021). EpiDope: a deep neural network for linear B-cell epitope prediction. Bioinformatics 37, 448–455. doi: 10.1093/bioinformatics/btaa773, PMID: 32915967

[ref6] Cramer P. (2021). AlphaFold2 and the future of structural biology. Nat. Struct. Mol. Biol. 28, 704–705. doi: 10.1038/s41594-021-00650-1, PMID: 34376855

[ref7] Du Z. Su H. Wang W. Ye L. Wei H. Peng Z. . (2021). The trRosetta server for fast and accurate protein structure prediction. Nat. Protoc. 16, 5634–5651. doi: 10.1038/s41596-021-00628-9, PMID: 34759384

[ref8] El-Manzalawy Y. Dobbs D. Honavar V. (2008a). Predicting linear B-cell epitopes using string kernels. J Mol Recognit. 21, 243–255. doi: 10.1002/jmr.893, PMID: 18496882 PMC2683948

[ref9] El-Manzalawy Y. Dobbs D. Honavar V. (2008b). Predicting flexible length linear B-cell epitopes. Comput. Syst. Bioinformatics (World Scientific) 7, 121–132. doi: 10.1142/9781848162648_0011 PMC3400678 19642274

[ref10] El-Manzalawy Y. Dobbs D. Honavar V. G. (2017). In silico prediction of linear B-cell epitopes on proteins. Methods Mol. Biol. 1484, 255–264. doi: 10.1007/978-1-4939-6406-2_17, PMID: 27787831 PMC8109234

[ref11] El-Manzalawy Y. Honavar V. (2010). Recent advances in B-cell epitope prediction methods. Immunome Res. 6, S2–S9. doi: 10.1186/1745-7580-6-S2-S2, PMID: 21067544 PMC2981878

[ref12] Guo M.-H. Liu Z.-N. Mu T.-J. Hu S.-M. (2022). Beyond self-attention: external attention using two linear layers for visual tasks. IEEE Trans. Pattern Anal. Mach. Intell. 14, 1–13. doi: 10.1109/TPAMI.2022.3211006, PMID: 36197869

[ref13] Hasan M. M. Khatun M. S. Kurata H. (2020). iLBE for computational identification of linear B-cell epitopes by integrating sequence and evolutionary features. Genom. Proteom. Bioinform. 18, 593–600. doi: 10.1016/j.gpb.2019.04.004, PMID: 33099033 PMC8377379

[ref14] He K. Zhang X. Ren S. Sun J. (2015). Spatial pyramid pooling in deep convolutional networks for visual recognition. IEEE Trans. Pattern Anal. Mach. Intell. 37, 1904–1916. doi: 10.1109/TPAMI.2015.2389824, PMID: 26353135

[ref15] Hinton G. E. Srivastava N. Krizhevsky A. Sutskever I. Salakhutdinov R. R. (2012). Improving neural networks by preventing co-adaptation of feature detectors. arXiv. arXiv:1207.0580 [Epub ahead of preprint]. doi: 10.48550/arXiv.1207.0580

[ref16] Hochreiter S. Schmidhuber J. (1997). Long short-term memory. Neural Comput. 9, 1735–1780. doi: 10.1162/neco.1997.9.8.1735 9377276

[ref17] Huang J. Honda W. (2006). CED: a conformational epitope database. BMC Immunol. 7, 1–8. doi: 10.1186/1471-2172-7-7, PMID: 16603068 PMC1513601

[ref18] Jespersen M. C. Peters B. Nielsen M. Marcatili P. (2017). BepiPred-2.0: improving sequence-based B-cell epitope prediction using conformational epitopes. Nucleic Acids Res. 45, W24–W29. doi: 10.1093/nar/gkx346, PMID: 28472356 PMC5570230

[ref19] Jumper J. Evans R. Pritzel A. Green T. Figurnov M. Ronneberger O. . (2021). Highly accurate protein structure prediction with AlphaFold. Nature 596, 583–589. doi: 10.1038/s41586-021-03819-2, PMID: 34265844 PMC8371605

[ref20] Khan A. Sohail A. Zahoora U. Qureshi A. S. (2020). A survey of the recent architectures of deep convolutional neural networks. Artif. Intell. Rev. 53, 5455–5516. doi: 10.1007/s10462-020-09825-6

[ref21] Krizhevsky A. Sutskever I. Hinton G. E. (2017). Imagenet classification with deep convolutional neural networks. Commun. ACM 60, 84–90. doi: 10.1145/3065386

[ref22] Larsen J. E. P. Lund O. Nielsen M. (2006). Improved method for predicting linear B-cell epitopes. Immunome Res. 2, 1–7. doi: 10.1186/1745-7580-2-2, PMID: 16635264 PMC1479323

[ref23] Li W. Godzik A. (2006). Cd-hit: a fast program for clustering and comparing large sets of protein or nucleotide sequences. Bioinformatics 22, 1658–1659. doi: 10.1093/bioinformatics/btl158 16731699

[ref24] Liu T. Shi K. Li W. (2020). Deep learning methods improve linear B-cell epitope prediction. BioData Mining. 13, 1–13. doi: 10.1186/s13040-020-00211-0, PMID: 32699555 PMC7371472

[ref25] Manavalan B. Govindaraj R. G. Shin T. H. Kim M. O. Lee G. (2018). iBCE-EL: a new ensemble learning framework for improved linear B-cell epitope prediction. Front. Immunol. 9:1695. doi: 10.3389/fimmu.2018.01695, PMID: 30100904 PMC6072840

[ref26] Murphy K. Weaver C. (2012). “The induced responses of innate immunity” in Janeway's Immunobiology. 8th *ed* eds. Scobie J. Lawrence E. Moldovan J. Lucas G. Goatly B. Toledo M. (New York, NY: Garland Science), 75–125.

[ref27] Peng L. Wang F. Wang Z. Tan J. Huang L. Tian X. . (2022). Cell–cell communication inference and analysis in the tumour microenvironments from single-cell transcriptomics: data resources and computational strategies. Brief. Bioinform. 23:bbac234. doi: 10.1093/bib/bbac234, PMID: 35753695

[ref28] Raffel C. Ellis D. P. (2015). Feed-forward networks with attention can solve some long-term memory problems. arXiv preprint arXiv:1512.08756 [Epub ahead of preprint]. doi: 10.48550/arXiv.1512.08756

[ref29] Ras-Carmona A. Pelaez-Prestel H. F. Lafuente E. M. Reche P. A. (2021). BCEPS: a web server to predict linear B cell epitopes with enhanced immunogenicity and cross-reactivity. Cells 10:2744. doi: 10.3390/cells10102744, PMID: 34685724 PMC8534968

[ref30] Schuster M. Paliwal K. K. (1997). Bidirectional recurrent neural networks. IEEE Trans. Signal Process. 45, 2673–2681. doi: 10.1109/78.650093

[ref31] Sharma S. Vashisht S. Gaur S. N. Lavasa S. Arora N. (2021). Identification of B cell epitopes of per a 5 allergen using bioinformatic approach. Immunobiology 226:152146. doi: 10.1016/j.imbio.2021.152146, PMID: 34717182

[ref32] Sharon J. Rynkiewicz M. J. Lu Z. Yang C. Y. (2014). Discovery of protective B-cell epitopes for development of antimicrobial vaccines and antibody therapeutics. Immunology 142, 1–23. doi: 10.1111/imm.12213, PMID: 24219801 PMC3992043

[ref33] Shen L. Liu F. Huang L. Liu G. Zhou L. Peng L. (2022). VDA-RWLRLS: an anti-SARS-CoV-2 drug prioritizing framework combining an unbalanced bi-random walk and Laplacian regularized least squares. Comput. Biol. Med. 140:105119. doi: 10.1016/j.compbiomed.2021.105119, PMID: 34902608 PMC8664497

[ref34] Shirai H. Prades C. Vita R. Marcatili P. Popovic B. Xu J. . (2014). Antibody informatics for drug discovery. Biochim Biophys Acta 1844, 2002–2015. doi: 10.1016/j.bbapap.2014.07.006 25110827

[ref35] Silver D. Schrittwieser J. Simonyan K. Antonoglou I. Huang A. Guez A. . (2017). Mastering the game of go without human knowledge. Nature 550, 354–359. doi: 10.1038/nature24270, PMID: 29052630

[ref36] Singh H. Ansari H. R. Raghava G. P. S. (2013). Improved method for linear B-cell epitope prediction using antigen’s primary sequence. PLoS One 8:e62216. doi: 10.1371/journal.pone.0062216, PMID: 23667458 PMC3646881

[ref37] Tian G. Wang Z. Wang C. Chen J. Liu G. Xu H. . (2022). A deep ensemble learning-based automated detection of COVID-19 using lung CT images and vision transformer and ConvNeXt. Front. Microbiol. 13:1024104. doi: 10.3389/fmicb.2022.1024104, PMID: 36406463 PMC9672374

[ref38] Van der Maaten L. Hinton G. (2008). Visualizing Data using t-SNE. J. Mach. Learn. Res. 9, 2579–2605.

[ref39] Vaswani A. Shazeer N. Parmar N. Uszkoreit J. Jones L. Gomez A. N. . (2017). “Attention is all you need” in Advances in Neural Information Processing Systems eds. Guyon I. Von Luxburg U. Bengio S. Wallach H. Fergus R. Vishwanathan S. Garnett R. Neural Information Processing Systems Foundation, Inc. (NeurIPS).

[ref40] Vita R. Mahajan S. Overton J. A. Dhanda S. K. Martini S. Cantrell J. R. . (2019). The immune epitope database (IEDB): 2018 update. Nucleic Acids Res. 47, D339–D343. doi: 10.1093/nar/gky1006, PMID: 30357391 PMC6324067

[ref41] Vita R. Overton J. A. Greenbaum J. A. Ponomarenko J. Clark J. D. Cantrell J. R. . (2015). The immune epitope database (IEDB) 3.0. Nucleic Acids Res. 43, D405–D412. doi: 10.1093/nar/gku938, PMID: 25300482 PMC4384014

[ref42] Vita R. Zarebski L. Greenbaum J. A. Emami H. Hoof I. Salimi N. . (2010). The immune epitope database 2.0. Nucleic Acids Res. 38, D854–D862. doi: 10.1093/nar/gkp1004, PMID: 19906713 PMC2808938

[ref43] Wang T. Wu D. J. Coates A. Ng A. Y. (2012). "End-to-end text recognition with convolutional neural networks," in *Proceedings of the 21st International Conference on Pattern Recognition (IEEE)*, pp. 3304–3308.

[ref44] Xu H. Zhao Z. (2022). NetBCE: an interpretable deep neural network for accurate prediction of linear B-cell epitopes. bioRxiv [Epub ahead of preprint]. doi: 10.1101/2022.05.23.493092 PMC10025766 36526218

[ref45] Yao B. Zhang L. Liang S. Zhang C. (2012). SVMTriP: a method to predict antigenic epitopes using support vector machine to integrate tri-peptide similarity and propensity. PLoS One 7:e45152. doi: 10.1371/journal.pone.0045152, PMID: 22984622 PMC3440317

[ref46] Zhang W. Xiong Y. Zhao M. Zou H. Ye X. Liu J. (2011). Prediction of conformational B-cell epitopes from 3D structures by random forests with a distance-based feature. BMC Bioinform. 12, 1–10. doi: 10.1186/1471-2105-12-341, PMID: 21846404 PMC3228550

